# A Case of Pyramidal Cataract

**Published:** 1863-07

**Authors:** E. S. Holmes

**Affiliations:** Chicago; One of the Surgeons of the Chicago Charitable Eye and Ear Infirmary


MEDICAL JOURNAL.

VOL. VI.] JULY, 1863. [No. 7.

### ORIGINAL COMMUNICATIONS.

### A CASE OF PYRAMIDAL CATARACT.

By E. L. HOLMES, M. D., of Chicago.
*One of the Surgeons of the Chicago Charitable Eye and Ear Infirmary.*

In January last, a young man, nineteen years of age, and apparently of a scrofulous diathesis, consulted me in reference to his left eye. I found the lids with evident signs of former inflammation, the conjunctiva being atrophied and replaced to a certain extent, by cartilagenous substance; there was also a tendency to trichi asis, although none of the cilia rested upon the cornea. In the centre of the cornea appeared a small opaque cicatrix, around which was a very narrow circle of extremely delicat and almost perfectly transparent nebulous deposit. From this central opacity ran two superficial vessels upwards to the conjunctiva. The other portions of the cornea were perfectly transparent. The whole capsule of the lens was opaque, as could be seen on dilating the pupil with atropine. The most remarkable feature of the case was the presence of a pyramid or cone of opaque substance, with its base attached to the lens and filling the pupil (when contracted by light), and its apex attached to the central cicatrix, of the cornea.

The base of this cone appeared to be a little less than a line in diametre. The distance from the pupil to the cornea might be possibly about two lines. The two superficial vessels mentioned above seemed to enter the anterior chamber through the cicatrix, and pass along the upper and outer side of the pyramid, into the lens. Whether the dark lines along the side of the cone were vessels, I am unable to decide positively. A single fact, however, which I will soon mention, inclines me to the opinion that they were. There was reason for believing the iris must necessarily be attached to the lens. But on introducing a few drops of solution of sulphate of atropia under the lids, the pupil became *fully and regularly dilated.* The presence of a few minute dark spots on the lens near the base of the cone, resembling detached portions of pigment from the iris, would seem to indicate that there had been slight adhesions, which the ordinary motions of the muscular fibres of the iris had detached.

The patient gave the following history of his case. At the age of three years he was attacked with inflammation of the eyes, which rendered the left eye almost useless and permanently diminished, to a considerable extent, the vision of the right one. The latter, seven years after, was totally destroyed by inflammation. When he was eight years of age, it was discovered that the left eye was affected with cataract. The lids were still somewhat granulated, and the cornea vascular; but they continued to improve in appearance, being subject, however, at intervals, to slight increase of congestion. The patient was considered incurable, and at the age of fourteen was placed for three years in the Michigan Institution for the Education of the Blind. About two years since, the presence of the pyramid connecting the lens and cornea, was observed. How long it had existed was a matter of doubt to physicians who had been able from time to time to examine the eye.

I am disposed to believe that during the first attack of inflammation, a small central ulcer perforated the cornea and caused the escape of the aqueous humor. The iris and lens were consequently brought in contact with the cornea. As the ulcer healed, a firm union between the cornea and centre of the lens took place, which prevented further escape of the aqueous humor. As the anterior chamber became filled, the iris and lens were restored to their normal position. It can be readily understood, as the lens receded, how the capsule must naturally be put upon the stretch and drawn out into the form of a pyramid. The substance of the pyramid was undoubtedly to a great extent composed of lymph, since, although the central portion of the capsule seemed drawn forward, the pyramid itself seemed to be some foreign body fastened upon the capsule. We believe such an adhesion as above described could seldom occur without involving a portion at least of the pupilary edge of the iris.

As the patient was totally blind, an operation seemed perfectly justifiable, although the condition of the lids and the tendency to conjestion from slight causes might possibly increase the danger of subsequent inflammation. At the patient's request, and with the advice of his friend and physician, Dr. Eugene Bitely, of Paw Paw, Michigan, I dilated the pupil widely with atropine and introduced a needle through the sclerotic. After depressing the needle from above downwards between the lens and iris, I carried the point into the anterior chamber nearly to the cornea and easily divided the cone as near its apex as possible. The needle had been passed betweent the lens and iris without the appearance of a drop of blood. At the moment of the division of the pyramid, however, a minute quantity of blood followed the point of the needle, which fact seems to prove that the superficial vessels of the cornea above mentioned really passed through the cornea into the substance of the pyramid and lens. As the needle was withdrawn, it was pressed through the capsule, when the anterior chamber was filled with a milky fluid and small fragments of the central portions of the cataract. After the operation the pupil was kept dilated with atropine, and fortunately, in a few days, the congestion produced by the operation partially subsided and the patient returned home.

A couple of weeks since I had an opportunity of examining the eye. The two vessels of the cornea had nearly disappeared; a very small portion of the apex of the pyramid could be seen projecting from the cornea into the anterior chamber. A small fragment of the cataract had fallen to the lower and outer portion of the anterior chamber. As the eye had entirely recovered from the effects of the operation, there seemed to be little reason to fear any harm from the presence of this portion of the capsule, especially as Dr. Bitely informed me it was gradually diminishing in size. The pupil was perfectly clear, and but for the presence of the central opacity of the cornea, the patient would undoubtedly see as well as any one could see, after an operation for cataract.

He was able to read the ordinary large letters on the title pages of books. At a distance of fifty or sixty rods he could distinguish the masts and rigging of vessels lying in the harbor. With the assistance of a double convex lens No. XX, he found distant objects much better defined, although no lens enabled him to read ordinary print. Undoubtedly an operation for artificial pupil or for removing the pupil to one side by iriddesis, would greatly improve vision. As the patient was already so well pleased with the result of the operation, and could conduct himself without difficulty wherever he chose to go, it was thought best to leave the eye for a time in its present condition.

Pyramidal Cataract, especially in the form above described, is a rare form of disease. In the eye clinics of Paris and Vienna, which I attended faithfully for nearly two years, I never observed a case similar in any respect to the one here reported. In my own private practice, with more than fifteen hundred registered cases, I have seen nothing analagous to it. I may, however, with propriety, perhaps, refer to the case of an Irish woman, aged thirty-three years, who consulted me in reference to one of her eyes. In the very centre of the cornea was an extremely minute white opacity, appearing as if a small particle of carbonate of lead had been involved in the cicatrix of a former ulcer. In the centre of the pupil, also, on the anterior capsule of the lens, was a spot precisely of the same description. The patient could give me no satisfactory account of the case, but informed me that in infancy she suffered from a severe attack of "sore eyes." Probably a perforating ulcer of the cornea caused the evacuation of the anterior chamber, when the centre of the capsule of the lens came in contact with the cornea. A small deposit of lymph was formed upon the capsule of the lens, without producing, however, a firm adhesion. As the ulcer healed, the lens was restored to its natural position, with no trace of disease remaining in the eye, except the minute opaque spot on the lens and cornea.

In this manner, we believe, nearly all the so called central anterior capsular cataracts are formed.

